# Mechanical load monitoring in rugby: limitations and future perspectives

**DOI:** 10.3389/fspor.2026.1840846

**Published:** 2026-06-08

**Authors:** Cristian Osgnach, Pietro E. di Prampero, Alberto Botter, Marco Maniera, Jean-Benoit Morin, Gaspare Pavei

**Affiliations:** 1 Department of Sport Science, Exelio srl, Udine, Italy; 2 Emeritus Professor of Physiology, University of Udine, Udine, Italy; 3 Sport Science Department, Benetton Rugby, Treviso, Italy; 4 University Jean Monnet Saint-Etienne, Lyon 1, University Savoie Mont-Blanc, Inter-University Laboratory of Human Movement Biology, Saint-Etienne, France; 5 Sport Science (STAPS) Department, Université Jean Monnet Saint-Etienne, Saint-Etienne, France; 6 Department of Pathophysiology and Transplantation, University of Milan, Milan, Italy

**Keywords:** acceleration, acceleration-speed profile, mechanical power, performance analysis (PA), rugby

## Abstract

**Purpose:**

Quantifying mechanical work in rugby is essential for improving performance, managing fatigue, and informing load monitoring practices. Traditional metrics that assess acceleration and speed separately fail to capture the interaction between these parameters during gameplay. This study presents an external power approach based on individual acceleration–speed profiles (ASP) to provide an integrated description of players' external work.

**Methods:**

Data were collected from 51 professional rugby matches across two seasons. Traditional metrics and external power indicators derived from ASPs were examined to evaluate mechanical demands. This approach combines acceleration and speed into a unified framework, enabling an individualised analysis of high external power actions.

**Results:**

Mechanical indicators showed differences between positional groups, with backs exhibiting higher values of high external power actions and external work than forwards. Traditional acceleration thresholds appeared to misclassify some high-demand efforts, particularly those initiated at higher running speeds. The external power approach provided an alternative representation of high-intensity locomotor demands by accounting for the interaction between speed and acceleration.

**Conclusion:**

The external power approach represents an additional framework for assessing running-related player-specific mechanical load in rugby, addressing limitations of traditional metrics based on fixed thresholds. It may support a more contextualised interpretation of high-intensity locomotor efforts and should be considered complementary to existing GPS-derived indicators. Future research should investigate its relationship with physiological and performance outcomes, as well as its integration with contact-based demands in rugby.

## Introduction

Defining the mechanical load imposed on the players is crucial for managing individual workloads. Mechanical load is often viewed as a range of demanding activities from a neuro-muscular and/or musculo-tendinous perspective that can usually be described via widespread parameters. High-speed running, accelerations, and decelerations are the most common and can be easily derived thanks to the data obtained from any accurate tracking technologies (i.e., GNSS and LPS wearable devices or video-based systems) (1–4). Although they are in widespread use, these variables are limited mainly because they treat running speed and acceleration separately, even though they occur concurrently during on-field activities and are mutually dependent. This may lead to a misrepresentation of the true external mechanical load experienced during match play. For this reason, given the interplay between speed and acceleration, an approach that considers both together would be preferable, and the use of power, whether metabolic (5–7) or mechanical (8, 9), may provide a viable solution. An additional group of activities should also be considered to identify the most intense phases in terms of external load in rugby. In fact, the majority of contact and set-piece periods (such as scrums, lineouts, rucks, or mauls) play an important role in rugby due to their high power outputs and should be included in the most demanding exercises (10, 11). However, today, it is not easy to detect them thanks to the sole use of technology (12, 13), and it's even more difficult to assess the actual forces acting on the players in both laboratory and field settings (14–16). Indeed, match analysts often code and count them as an additional arbitrary work; yet they engage the players' neuro-muscular and locomotor systems, albeit in a way that differs from running actions. Despite the difficulties in assessing high-intensity locomotion using traditional parameters, the aim of the present study is to describe match demands while also employing the external power approach (8, 9, 17), which should help to overcome the aforementioned limitation. Although we are fully aware that the decelerations play a significant role in the physiological demands from a neuromuscular perspective, this manuscript concentrates on the positive phases with the aim of establishing a standardised analysis of accelerations in general. After describing the external power approach, official match data from a professional rugby team will be presented and discussed.

However, despite these advances, the application of an individualised acceleration-speed framework, combined with external power, to describe match demands in professional rugby remains limited, particularly in ecologically valid match settings.

In this context, despite their widespread use, acceleration-count–based methods often fail to accurately identify high-mechanical-load events, particularly when actions are initiated at different running speeds. The present study addresses this limitation by proposing a model that provides a more realistic and integrated identification of such events through the combined assessment of speed and acceleration.

## External power approach: a two-step procedure

**Figure 1:**
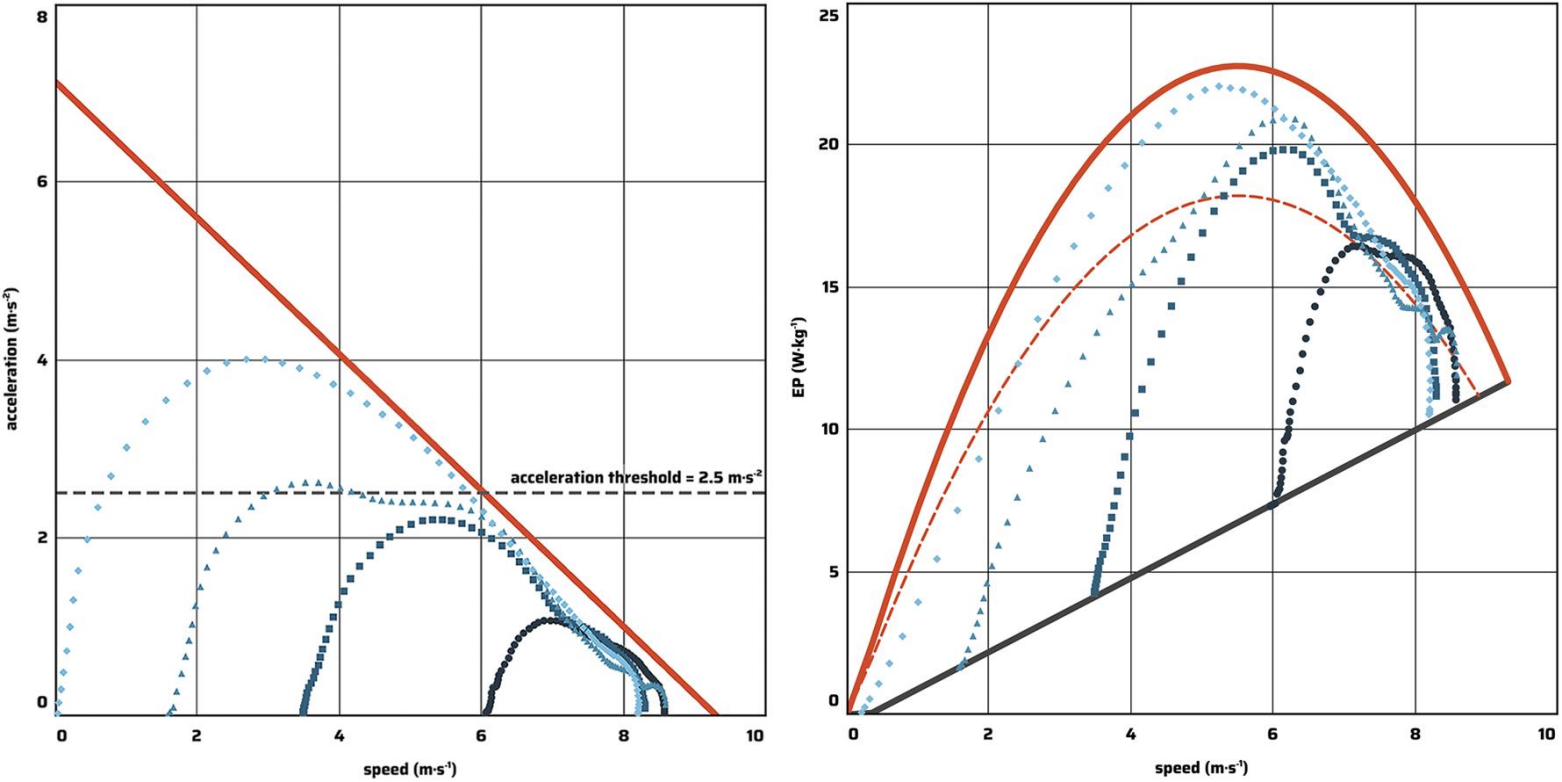
Left panel: the acceleration-speed profile (ASP) is the relationship between acceleration (*Y*-axis) and speed (*X*-axis). Right panel: the corresponding external power (EP) is obtained from the appropriate ASP speed and acceleration values (thick red curve); eighty percent of the EP calculated over the entire speed spectrum is used as a power limit to detect activities performed at higher intensities (dashed thin red curve).

The mechanical power approach allows us to overcome the flaws of a fixed-acceleration-threshold criterion. In fact, isolating the most intense activities by using acceleration alone would not be appropriate, since the intensity of the chosen acceleration threshold is strictly related to the corresponding running speed, as illustrated by the individual acceleration-speed profile (ASP) (18, 19). In other words, a specific acceleration value (for example, 2.5 m⋅s⁻^2^, one of the most frequently used thresholds for calculating the number of acceleration events) can be (i) only a fraction of the maximum acceleration achievable from a standing start, (ii) the acceleration peak when the player starts sprinting from a certain moderate speed, or (iii) unobtainable acceleration if the athlete is already running quite fast (20). Furthermore, attaining the same level of acceleration (2.5 m⋅s⁻^2^) at various speeds requires different resources and imposes significantly different “loads” on the player's system. For this purpose, ASP can be used as a reference, allowing us to define, for each individual, the interplay between running speed (from 0 to top speed) and the corresponding achievable acceleration (see Figure 1, left panel). Once the player's ASP is obtained through a maximal sprint test (18, 21) or the *in-situ* approach (22–25), the corresponding external power (EP) associated to running acceleration can be calculated across the entire speed spectrum (9, 17). Hence, a fraction of the maximum EP values can be used as the threshold to capture high external power actions (bursts), thus allowing actual speed and acceleration to be considered simultaneously (see Figure 1, right panel).

Setting this threshold represents the first step in the process, followed by assessing the external work done when the actual EP exceeds it (high external work, hEW) and counting the number of bursts. A further level of detail can be obtained by dividing the overall amount of hEW, expressed in J⋅kg⁻^1^, into its three components: (i) the activity performed at low speed with a prevalence of force (hEW_f_), (ii) the activity carried out at intermediate speed with a predominance of power (hEW_p_), and (iii) the remaining amount held at higher speed (hEW_s_) (for more details on this and related matters, see the following section Materials and Methods, and (9). The subsequent paragraphs are intended to present and discuss traditional and external power indicators related to official matches.

## Materials and methods

A professional rugby team competing in the United Rugby Championship (URC) tracked fifty-one official matches during the 2022–2023 and 2023–2024 seasons. A total of 1,172 match observations from 64 players across two seasons were considered. A match observation was defined as a single player participation in a match, regardless of playing time, corresponding to a player-match exposure. Players typically trained four sessions per week (Monday, Tuesday, Thursday, and Friday) and played official matches on Saturday, with Wednesday and Sunday generally as rest days (mean ± SD; age = 27.8 ± 4.0 yr; body mass = 104.0 ± 12.0 kg; stature = 1.85 ± 0.08 m).

**Figure 2:**
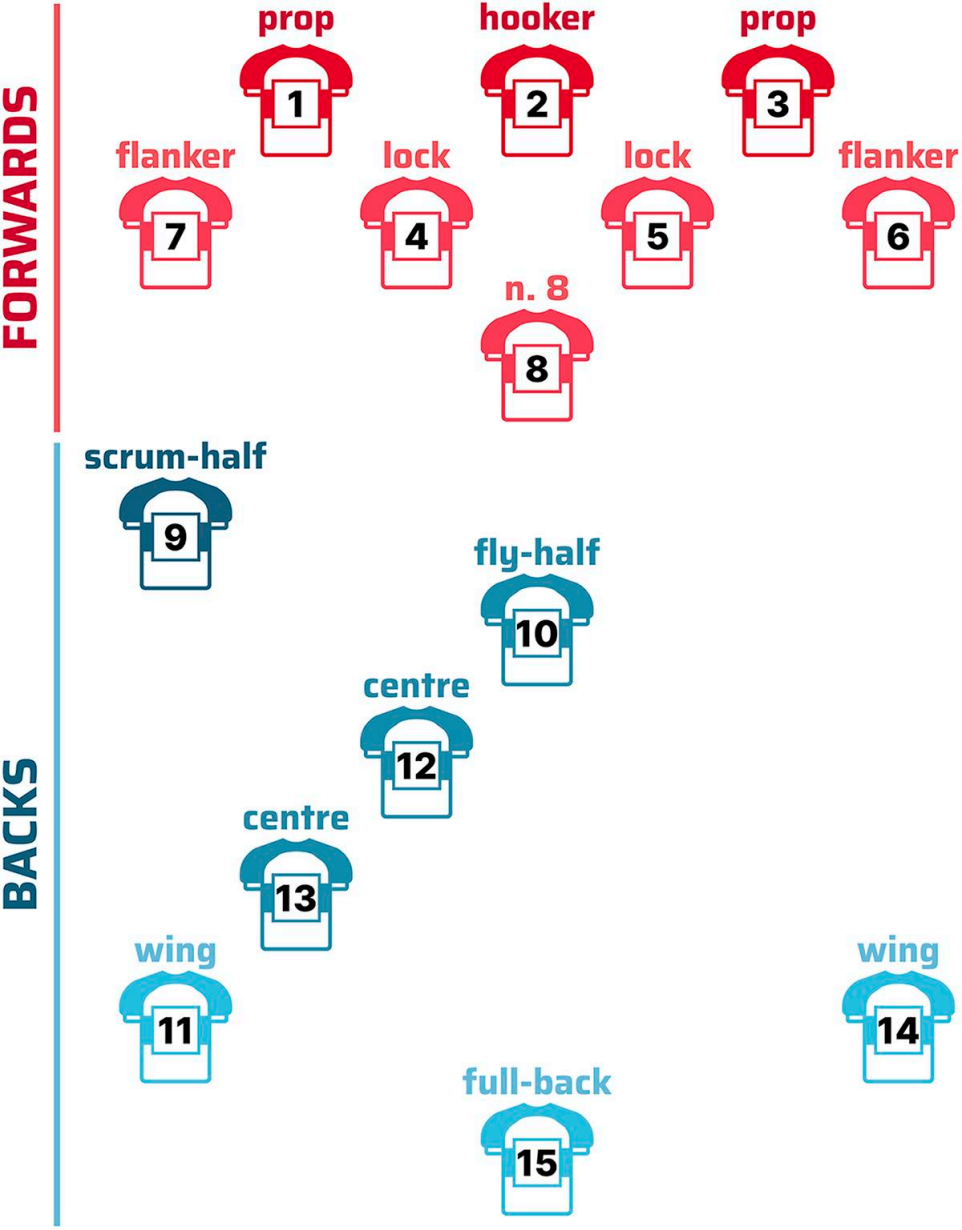
Team formation: players' positions were divided into two main categories: Forwards (red) and Backs (blue). Each category was further split into positional groups: Props + Hooker (dark red), and Locks + Flankers + Number 8 (light red) belonging to Forwards; Number 9 (dark blue), Fly-Halfs + Centers (blue), and Wings + Fullback (light blue) belonging to Backs.

Players were categorised as follows (Figure 2): Forwards (*n* = 39; match observations = 665), including Props and Hooker (*n* = 17; match observations = 295), and Locks, Flankers, and Number 8 (*n* = 22; match observations = 370); Backs (*n* = 42; match observations = 507), including Number 9 (*n* = 6; match observations = 92), Fly-Halfs and Centers (*n* = 18; match observations = 227), and Wings and Fullback (*n* = 18; match observations = 188). Players' movements were tracked using GPS units (gpexe Pro^2^, 18.18 Hz, firmware v. 78, Exelio srl, Udine, Italy) worn in a pocket of a specific vest located on the back between the shoulders and processed using the proprietary software (gpexe web app v. 7.5). Previous research has demonstrated the reliability and concurrent validity of the GPS system used (26).

### Acceleration-speed profiles

Individual *in-situ* ASPs were computed monthly via the GPS software, applying the procedure from Morin et al. (24) and considering all matches and training sessions completed by each player during this period. In addition to the typical profile data, namely maximum theoretical speed (S₀, m⋅s⁻^1^), maximum theoretical acceleration (A₀, m⋅s⁻^2^), and time constant (*τ*, s), the number of sessions used (N_ASP_, #) and the corresponding overall duration (T_ASP_, min) were also determined.

### Locomotion indicators

Traditional locomotion parameters obtained from GPS included total distance covered (TD, m), corresponding average speed (s̄, m⋅min⁻^1^), distance covered above 5.5 m⋅s⁻^1^ (D_s_ _>_ _5.5_, m) and 7.0 m⋅s⁻^1^ (D_s_ _>_ _7.0_, m). In addition, the number of speed events above 5.5 m⋅s⁻^1^ (N_s_ _>_ _5.5_, #) and accelerations above 2.5 m⋅s⁻^2^ for a minimum time greater than 0.5 s (N_a_ _>_ _2.5_, #) was determined. Since nearly all substitutions involve replacing one player with another in the same position, a *per-individual field position* average (9) was calculated by dividing the total from all players who participated in the match by the number of players occupying that position in the base lineup (see Figure 2).

### External power indicators

Similar to locomotion parameters, the corresponding mechanical analysis yielded total external work associated with running speed and acceleration (EW, J⋅kg⁻^1^), related external power (EP, W⋅kg⁻^1^), and external work done above 80% of the maximal power output over the entire speed spectrum (hEW, J⋅kg⁻^1^). The 80% threshold was adopted in line with previous work on mechanical power in team sports, where relative intensity cut-offs of individual maximal external power are used to identify the upper tail of high-intensity locomotor actions (8, 9). hEW was divided into three components based on speed criteria: external work achieved with a prevalence of force (hEW_f_, J⋅kg⁻^1^) in a speed range from 0 to 1/3 S₀; with a dominance of power (hEW_p_, J⋅kg⁻^1^) between 1/3 S₀ and 2/3 S₀; and work done at speeds higher than 2/3 S₀ (hEW_s_, J⋅kg⁻^1^). The number of high external power events (bursts, #) was also obtained (see the preceding section, External Power Approach, for more details).

The data for this retrospective analysis were collected during official matches, encompassing standard technical and medical oversight, without any experimental intervention or modification of the athletes' regular activities. Moreover, all data were anonymised and did not include personally identifiable information. Results are shown as means ± SD, unless noted otherwise. Data were analysed at the population level and are presented as descriptive statistics and linear trends over playing time. No formal inferential statistical comparisons were conducted between positional groups; therefore, the results should be interpreted as descriptive differences in group-level trends.

## Results

The mean official match time was 89 min 49 s ± 6 min 47 s. Table 1 presents the team performance indicators *as a whole* for both locomotor and external power approaches.

**Table 1:** Average locomotor and mechanical indicators of the whole team (means + SD) and corresponding minimum and maximum values (in brackets) obtained from 51 official matches.

Locomotion	Mechanical
TD (m) 97,177 ± 7,101 (86,369–108,345)	EW (J⋅kg⁻^1^) 90,685 ± 7,485 (78,175–101,781)
s̄ (m⋅min⁻^1^) 71.4 ± 4.4 (62.6–78.1)	EP (W⋅kg⁻^1^) 1.11 ± 0.09 (0.95–1.24)
D_s_ _>_ _5.5_ (m) 4,983 ± 876 (3,518–6,366)	hEW (J⋅kg⁻^1^) 2,819 ± 497 (2,133–3,659)
D_s_ _>_ _7.0_ (m) 1,074 ± 258 (732–1,505)	hEW_f_ (J⋅kg⁻^1^) 656 ± 115 (451–879)
N_s_ _>_ _5.5_ (#) 333 ± 51 (254–415)	hEW_p_ (J⋅kg⁻^1^) 1,344 ± 268 (946–1,875)
N_a_ _>_ _2.5_ (#) 645 ± 75 (530–762)	hEW_s_ (J⋅kg⁻^1^) 819 ± 223 (497–1,165)
	bursts (#) 280 ± 38 (207–341)

**Locomotion abbreviations**: TD, total distance; s̄, average speed; D_s_ _>_ _5.5_, distance covered above 5.5 m⋅s⁻^1^; D_s_ _>_ _7.0_, distance covered above 7.0 m⋅s⁻^1^; N_s_ _>_ _5.5_, number of high-speed events above 5.5 m⋅s⁻^1^; N_a_ _>_ _2.5_, number of acceleration events above 2.5 m⋅s⁻^2^.

**External power abbreviations**: EW, external work; EP, external power; hEW, external work done above 80% of the maximal power output; hEW_f_, external work done above 80% of the maximal power output when the speed is between 0 and 1/3 S₀; hEW_p_, external work done above 80% of the maximal power output when the speed is between 1/3 S₀ and 2/3 S₀; hEW_s_, external work done above 80% of the maximal power output when the speed is between 2/3 S₀ and S₀; bursts, number of mechanical events above 80% of the maximal power output.

### Acceleration-speed profiles

**Figure 3:**
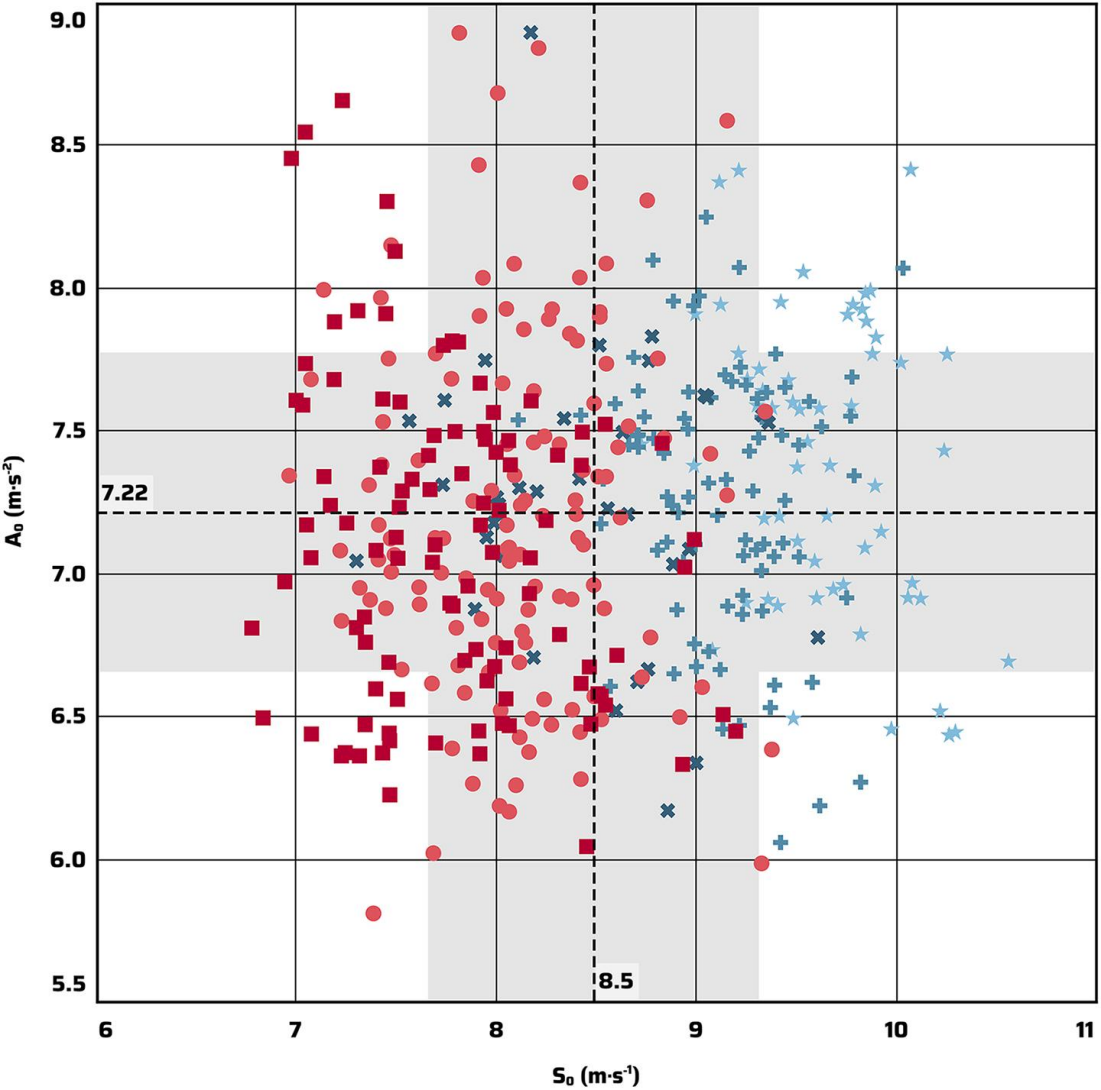
Monthly *in-situ* ASP values of all individual players (S₀ and A₀); Forwards (red) and Backs (blue) are further detailed in positional groups: Props + Hooker (dark red, square), Locks + Flanker + Number 8 (light red, circle), number 9 (dark blue, cross), Fly-Halfs + Centers (blue, plus), and Wings + Fullback (light blue, star). Means and SD are indicated for each axis by the black dashed lines and the grey bands, respectively.

A total of 524 monthly *in-situ* ASPs were processed, which included all the training and official match sessions completed by the active players over the two seasons analysed. Any profile obtained by a player involved in at least one return-to-play session during the reference month was excluded. The inclusion criteria to enable an objective evaluation of the robust ASPs (23, 27) were as follows: (i) at least nine training or match sessions within the month (N_ASP_ ≥ 9) for a total of (ii) at least 480 working minutes (T_ASP_ ≥ 480). Thus, the remaining profiles that met these requirements totalled 414 *in-situ* ASPs derived from 64 different players (Figure 3). For each season, the average S₀ and A₀ of all ASPs belonging to a single athlete were used to calculate the external power indicators. Table 2 presents a summary of the ASPs obtained and averaged by role.

**Table 2:** Average ASPs characteristics (S₀, maximum theoretical speed; A₀, maximum theoretical acceleration; *τ*, time constant) for each positional group (mean ± SD).

ASP KPIs	Forwards		Backs		
	Props Hooker	Locks Flankers Number 8	Number 9	Fly-Halfs Centers	Wings Fullback
	(*N* = 103)	(*N* = 127)	(*N* = 37)	(*N* = 85)	(*N* = 62)
S₀ (m⋅s⁻^1^)	7.78 ± 0.53	8.11 ± 0.47	8.42 ± 0.56	9.14 ± 0.36	9.64 ± 0.39
A₀ (m⋅s⁻^2^)	7.09 ± 0.55	7.18 ± 0.63	7.26 ± 0.50	7.27 ± 0.46	7.38 ± 0.52
*τ* (s)	1.10 ± 0.13	1.14 ± 0.12	1.16 ± 0.12	1.26 ± 0.11	1.31 ± 0.13

### Locomotion indicators

**Figure 4:**
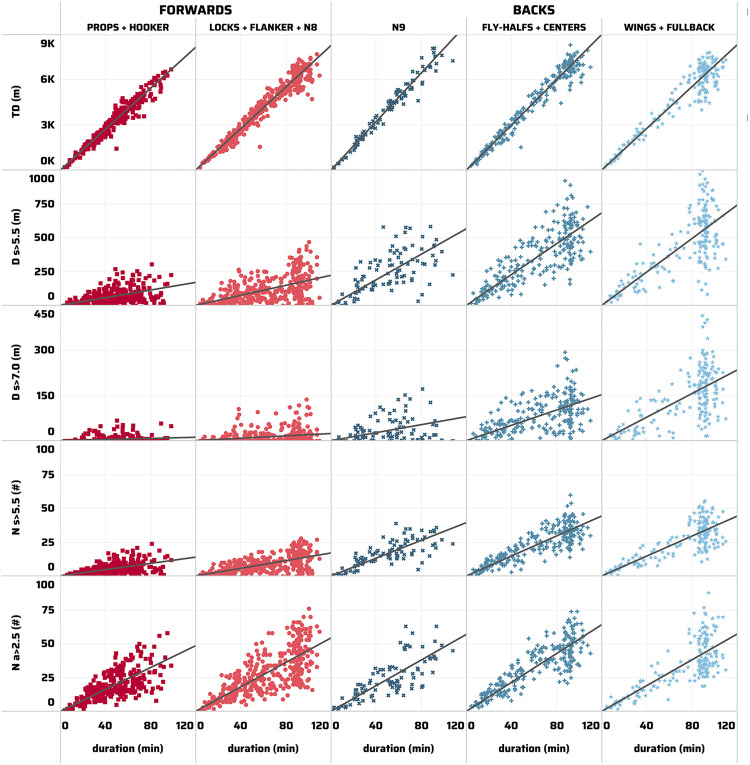
Locomotor indicators as a function of the playtime are reported for each positional group and interpolated by linear regressions (black lines). Forwards (red) and Backs (blue) are further detailed in positional groups: Props + Hooker (dark red, square), Locks + Flanker + Number 8 (light red, circle), Number 9 (dark blue, cross), Fly-Halfs + Centers (blue, plus), and Wings + Fullback (light blue, star).

Reporting the arithmetical average values for the match is not always helpful because only some players participating in the game complete it from start to finish. As such, it may also be interesting to represent all the indicators graphically as a function of playing time. This has a double advantage: (i) all the players can be included according to the time spent on the pitch, and (ii) the equation from the linear interpolation between time and each indicator, forcing the y-intercept to zero, can be considered a parameter of “amount per minute” to establish a reference value. Linear regressions were constrained to pass through the origin, assuming zero performance output at zero playing time. The linear time-based regressions should be interpreted as descriptors of workload accumulation per unit of match exposure rather than inferential models. The slope of each relationship represents the rate of external load accumulation over playing time, providing a practical metric of positional demands. Figure 4 and Table 3 show the values and equations for locomotor indicators.

**Table 3:** For each locomotor indicator, as illustrated in Figure 4, linear regression equations and R^2^ values are reported; the per-individual field position mean ± SD is reported, with the corresponding minimum and maximum values indicated in brackets.

Locomotion KPIs	Forwards		Backs		
	Props Hooker	Locks Flankers Number 8	Number 9	Fly-Halfs Centers	Wings Fullback
	(*N* = 295)	(*N* = 370)	(*N* = 92)	(*N* = 227)	(*N* = 188)
TD (m)	TD = 68·min R^2^ = .99 6,236 ± 393 (5,274–6,972)	TD = 69·min R^2^ = .99 6,318 ± 355 (5,530–6,936)	TD = 81·min R^2^ = .99 7,448 ± 567 (5,087–8,213)	TD = 75·min R^2^ = .99 6,910 ± 592 (5,233–8,283)	TD = 69·min R^2^ = .98 6,301 ± 534 (5,114–7,274)
s̄ (m⋅min⁻^1^)	68.5 ± 3.2 (60.5–74.2)	69.6 ± 3.1 (61.7–76.2)	81.5 ± 4.9 (71.5–90.0)	75.7 ± 4.3 (68.0–82.7)	70.0 ± 4.7 (60.4–78.3)
D_s_ _>_ _5.5_ (m)	D_s_ _>_ _5.5_ = 1.4·min R^2^ = .66 130.5 ± 37.4 (64.8–219.5)	D_s_ _>_ _5.5_ = 1.8·min R^2^ = .70 170.0 ± 29.2 (121.7–223.9)	D_s_ _>_ _5.5_ = 4.7·min R^2^ = .83 447.0 ± 134.7 (175.1–665.1)	D_s_ _>_ _5.5_ = 5.7·min R^2^ = .91 526.7 ± 104.7 (340.7–739.7)	D_s_ _>_ _5.5_ = 6.2·min R^2^ = .90 556.0 ± 77.0 (403.3–692.7)
D_s_ _>_ _7.0_ (m)	D_s_ _>_ _7.0_ = 0.1·min R^2^ = .16 7.9 ± 6.7 (0.0–31.6)	D_s_ _>_ _7.0_ = 0.2·min R^2^ = .27 17.5 ± 6.9 (6.4–32.2)	D_s_ _>_ _7.0_ = 0.7·min R^2^ = .48 63.0 ± 43.6 (2.7–168.9)	D_s_ _>_ _7.0_ = 1.3·min R^2^ = .76 121.1 ± 36.0 (55.1–191.6)	D_s_ _>_ _7.0_ = 2.0·min R^2^ = .83 175.1 ± 31.5 (109.4–247.1)
N_s_ _>_ _5.5_ (#)	N_s_ _>_ _5.5_ = 0.12·min R^2^ = .71 10.8 ± 2.6 (6–16)	N_s_ _>_ _5.5_ = 0.14·min R^2^ = .77 13.1 ± 2.2 (9–18)	N_s_ _>_ _5.5_ = 0.33·min R^2^ = .89 31.0 ± 7.1 (13–46)	N_s_ _>_ _5.5_ = 0.37·min R^2^ = .93 34.1 ± 5.8 (24–45)	N_s_ _>_ _5.5_ = 0.37·min R^2^ = .92 33.3 ± 4.6 (24–42)
N_a_ _>_ _2.5_ (#)	N_a_ _>_ _2.5_ = 0.40·min R^2^ = .87 37.6 ± 4.7 (27–45)	N_a_ _>_ _2.5_ = 0.45·min R^2^ = .88 41.6 ± 3.9 (35–50)	N_a_ _>_ _2.5_ = 0.47·min R^2^ = .89 43.8 ± 11.0 (22–63)	N_a_ _>_ _2.5_ = 0.53·min R^2^ = .94 50.5 ± 6.2 (38–63)	N_a_ _>_ _2.5_ = 0.48·min R^2^ = .91 43.0 ± 5.6 (32–53)

**Locomotion abbreviations**: TD, total distance; s̄, average speed; D_s_ _>_ _5.5_, distance covered above 5.5 m⋅s⁻^1^; D_s_ _>_ _7.0_, distance covered above 7.0 m⋅s⁻^1^; N_s_ _>_ _5.5_, number of high-speed events above 5.5 m⋅s⁻^1^; N_a_ _>_ _2.5_, number of acceleration events above 2.5 m⋅s⁻^2^.

### External power indicators

**Figure 5:**
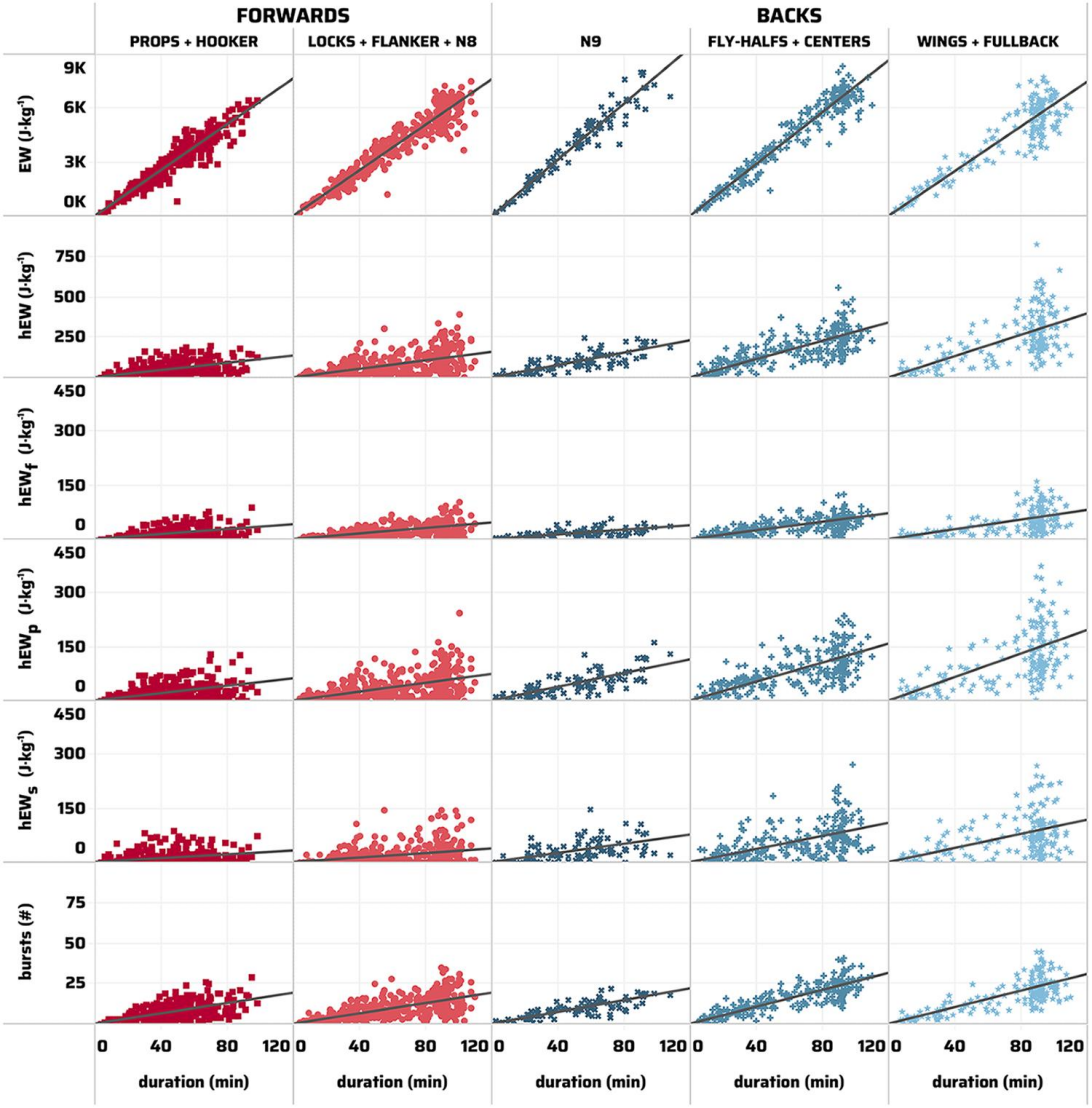
Mechanical indicators as a function of the playtime are reported for each positional group and interpolated by linear regressions (black lines). Forwards (red) and Backs (blue) are further detailed in positional groups: Props + Hooker (dark red, square), Locks + flanker + Number 8 (light red, circle), Number 9 (dark blue, cross), Fly-Halfs + Centers (blue, plus), and Wings + Fullback (light blue, star).

Similar to the previously established approach for locomotion indicators, the results in this section are also presented in the same format, with the KPIs illustrated in Figure 5 and Table 4.

**Table 4:** For each mechanical indicator, as illustrated in Figure 5, linear regression equations and R^2^ values are reported; the per-individual field position mean ± SD is reported, with the corresponding minimum and maximum values indicated in brackets.

External power KPIs	Forwards		Backs		
	Props Hooker	Locks Flankers Number 8	Number 9	Fly-Halfs Centers	Wings Fullback
	(*N* = 295)	(*N* = 370)	(*N* = 92)	(*N* = 227)	(*N* = 188)
EW (J⋅kg⁻^1^)	EW = 64·min R^2^ = .98 5,856 ± 470 (4,873–6,729)	EW = 63·min R^2^ = .99 5,821 ± 397 (4,933–6,637)	EW = 78·min R^2^ = .99 7,225 ± 675 (4,949–8,306)	EW = 72·min R^2^ = .99 6,640 ± 618 (5,215–8,075)	EW = 62·min R^2^ = .97 5,644 ± 573 (4,209–6,712)
EP (W⋅kg⁻^1^)	1.08 ± 0.06 (0.89–1.17)	1.07 ± 0.06 (0.95–1.21)	1.32 ± 0.11 (1.07–1.49)	1.22 ± 0.08 (1.03–1.35)	1.05 ± 0.08 (0.89–1.22)
hEW (J⋅kg⁻^1^)	hEW = 1.13·min R^2^ = .67 108.2 ± 23.8 (67.7–162.3)	hEW = 1.32·min R^2^ = .69 120.7 ± 23.0 (79.4–162.6)	hEW = 1.91·min R^2^ = .89 175.2 ± 41.9 (95.0–253.0)	hEW = 2.83·min R^2^ = .87 266.6 ± 56.0 (180.7–401.3)	hEW = 3.29·min R^2^ = .82 295.1 ± 53.5 (206.3–402.0)
hEW_f_ (J⋅kg⁻^1^)	hEW_f_ = 0.34·min R^2^ = .61 31.4 ± 6.8 (17.7–44.3)	hEW_f_ = 0.38·min R^2^ = .73 34.7 ± 5.6 (23.2–46.8)	hEW_f_ = 0.32·min R^2^ = .75 30.2 ± 10.0 (11.0–50.0)	hEW_f_ = 0.60·min R^2^ = .82 57.4 ± 13.6 (30.0–82.3)	hEW_f_ = 0.67·min R^2^ = .77 59.2 ± 9.8 (40.7–79.7)
hEW_p_ (J⋅kg⁻^1^)	hEW_p_ = 0.51·min R^2^ = .58 48.8 ± 11.7 (27.7–70.0)	hEW_p_ = 0.62·min R^2^ = .64 56.3 ± 11.7 (37.8–80.4)	hEW_p_ = 0.96·min R^2^ = .85 84.7 ± 23.3 (44.0–131.1)	hEW_p_ = 1.32·min R^2^ = .84 125.2 ± 28.2 (79.3–180.3)	hEW_p_ = 1.64·min R^2^ = .78 145.4 ± 28.6 (92.7–199.3)
hEW_s_ (J⋅kg⁻^1^)	hEW_s_ = 0.27·min R^2^ = .36 27.1 ± 13.2 (6.7–55.7)	hEW_s_ = 0.32·min R^2^ = .40 28.8 ± 9.9 (12.8–51.8)	hEW_s_ = 0.64·min R^2^ = .67 56.8 ± 27.1 (19.0–116.3)	hEW_s_ = 0.91·min R^2^ = .70 84.7 ± 27.1 (33.7–153.0)	hEW_s_ = 0.98·min R^2^ = .68 89.1 ± 28.2 (42.0–153.3)
bursts (#)	burst = 0.16·min R^2^ = .77 14.8 ± 2.3 (10–19)	burst = 0.16·min R^2^ = .79 14.7 ± 1.9 (11–18)	burst = 0.18·min R^2^ = .91 16.9 ± 3.7 (9–23)	burst = 0.26·min R^2^ = .92 24.6 ± 4.3 (16–33)	burst = 0.26·min R^2^ = .90 22.9 ± 3.1 (17–29)

**External power abbreviations**: EW, external work; EP, external power; hEW, external work done above 80% of the maximal power output; hEW_f_, external work done above 80% of the maximal power output when the speed is between 0 and 1/3 S₀; hEW_p_, external work done above 80% of the maximal power output when the speed is between 1/3 S₀ and 2/3 S₀; hEW_s_, external work done above 80% of the maximal power output when the speed is between 2/3 S₀ and S₀; bursts, number of mechanical events above 80% of the maximal power output.

## Discussion

The preceding sections were devoted to an analysis of the interactions between speed and acceleration, with the aim of assessing rugby players' mechanical work in greater detail. Focusing solely or separately on speed or acceleration has significant limitations, especially regarding acceleration. This is particularly evident in rugby, where (i) there are marked differences in roles, with larger athletes occupying forward positions (mass = 112.2 ± 5.7 kg, stature = 1.88 ± 0.07 m) compared to leaner players in the backline (mass = 92.8 ± 8.8 kg, stature = 1.81 ± 0.06 m). Additionally, (ii) compared to other team sports, which generally feature a more continuous style of play, rugby players often start from slower speeds, which facilitates the counting of a large number of accelerations—exceeding 2.5 m⋅s⁻^2^—that do not necessarily require significant effort, as previously highlighted by Osgnach et al. (9) in soccer players.

### Acceleration-speed profiles

Anthropometric differences between forwards and backs can also be highlighted through AS profiling (S₀ = 7.94 ± 0.56 m⋅s⁻^1^, A₀ = 7.14 ± 0.60 m⋅s⁻^2^ vs. S₀ = 9.17 ± 0.58 m⋅s⁻^1^, A₀ = 7.31 ± 0.49 m⋅s⁻^2^, respectively), especially concerning top-speed abilities.

Referring to ASP rather than a single, absolute acceleration threshold provides an alternative framework to interpret high external power bursts because (i) it represents an objective upper limit for each player, (ii) it can be periodically updated, based on easily accessible training and match data, to reflect each player's current condition throughout the season, and (iii) it allows accounting for the interaction between acceleration and the running speed at which it is produced, which is not considered when using acceleration alone (see Figure 1, left panel). Indeed, a fraction of the maximum external power yielded by the combinations of speed and acceleration from ASP can be used to identify high-intensity actions from a mechanical perspective (Figure 1, right panel). Finally, it is useful to point out that (iv) the *in-situ* procedure allows profiling players without requiring additional testing sessions, which may represent a practical advantage in applied settings. External power indicators calculated via ASP may therefore provide complementary—player-specific—information on high-intensity locomotor demands compared to traditional parameters. The following section aims to discuss this state of affairs in more detail.

### Locomotion vs. external power indicators

The main aim of this manuscript is to highlight the differences between traditional parameters used to describe mechanical load and those based on the external power approach. In this section, we would like to emphasise the limitations of acceleration events, which may not fully represent mechanical load when considered in isolation. In simple terms, overlooking the speed at which any acceleration starts, the intensity of that action cannot be appreciated by focusing solely on the acceleration. The main limitation of fixed acceleration thresholds lies in their inability to account for the speed-dependent nature of acceleration demands.

**Figure 6:**
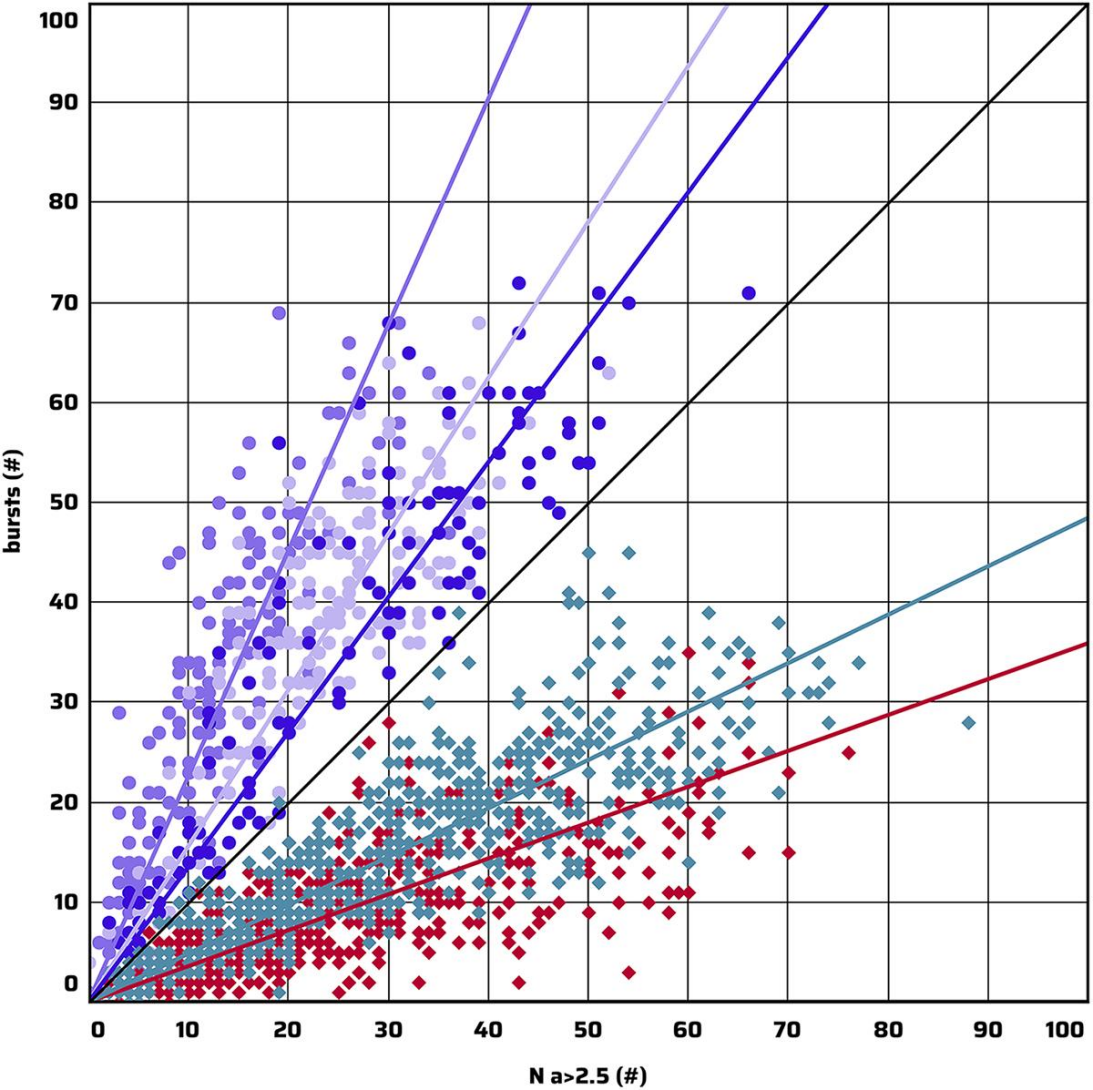
The number of bursts is plotted as a function of the number of accelerations > 2.5 m⋅s⁻^2^ (N_a_ _>_ _2.5_). Linear regressions for each positional group are also shown. Data from Forwards and Backs (red and blue diamonds, respectively) are presented alongside data from Osgnach et al. (9) regarding soccer Defenders, Midfielders, and Attackers (light, normal, and dark purple circles, respectively). The identity line (black) is also indicated.

In contrast to findings from soccer, where the number of bursts exceeded accelerations (9), the data analysed in rugby players show the opposite trend. The ratio between bursts and N_a_ _>_ _2.5_ for soccer players was 1.64, decreasing to 0.48 for backs and 0.36 for forwards in rugby players. These differences may be influenced by sport-specific characteristics, particularly the initial speed preceding accelerations, which appears to be higher in soccer. However, such cross-sport comparisons should be interpreted with caution due to structural and contextual differences between sports, including playing tempo, tactical organisation, and match dynamics. For instance, despite similar match durations (95:23 ± 01:58 in soccer vs. 89:49 ± 06:47 in rugby), the average speed observed in rugby players is lower (69.2 and 74.1 m⋅min⁻^1^ for forwards and backs) than in soccer players (111.3 m⋅min⁻^1^), suggesting that rugby players more frequently initiate movements from lower speeds. This characteristic may also be influenced by the high frequency of contact situations, such as tackles, rucks, and mauls, which repeatedly interrupt play and require players to restart movement from low or near-zero speeds. In addition, set-piece events such as scrums and lineouts, which can occur frequently during a match, further contribute to this pattern by requiring players to repeatedly initiate movement from (or near) zero speed. This may contribute to a higher number of acceleration events exceeding fixed thresholds, without necessarily reflecting comparable mechanical demands (Figure 6). This sport-specificity highlights that the nature of the bursts does not align with accelerations above a fixed threshold.

In addition to the number of occurrences, the amount of work performed at high external power output (hEW, J⋅kg⁻^1^) provides further details about exposure to higher mechanical loads. Backs behave more like attackers in soccer because the amount of external work carried out per individual burst is almost the same (11.2 vs. 11.8 J⋅kg⁻^1^ per burst). Conversely, forwards perform shorter actions because the external work done is lower compared to defenders and midfielders in soccer (7.8 vs. 9.9 J⋅kg⁻^1^ per burst).

The resulting framework, derived from the external power parameters, provides an alternative perspective on the differences between forwards and backs. Indeed, although forwards produced only 12% fewer N_a_ _>_ _2.5_ events than backs (0.44 vs. 0.50 N_a_ _>_ _2.5_⋅min⁻^1^), larger differences were observed when considering bursts (0.16 vs. 0.25 bursts⋅min⁻^1^) and hEW (1.26 vs. 2.94 hEW⋅min⁻^1^). These descriptive differences suggest that metrics that account for both speed and acceleration may offer additional insight into positional demands by capturing the interaction between speed and acceleration.

It is important to note that the differences reported above between forwards and backs pertain only to the locomotor component of match play. A major limitation of the present framework, as well as of the speed or acceleration approaches, is that it does not account for collisions, scrums, rucks, mauls, and other set-piece actions, which constitute a substantial component of total match demands in rugby. This limitation is particularly relevant for forwards, whose overall mechanical load is therefore likely underestimated when considering locomotor metrics alone (28).

From an applied perspective, the external power approach may provide additional insight for coaches and sport scientists by accounting for the interaction between speed and acceleration, which is not captured by fixed-threshold metrics. For instance, it may help differentiate between accelerations performed from low vs. high initial speeds, which may impose different mechanical demands despite similar acceleration values. This may support a more refined interpretation of training loads, helping practitioners to better contextualise high-intensity efforts across sessions and matches. Overall, the proposed framework should be viewed as complementary to existing velocity-based indicators, providing an additional layer of analysis—based on player-specific values—rather than replacing established metrics.

## Conclusion

This study highlights the potential of the external power approach as an additional framework for assessing running-related mechanical load in rugby players, addressing some of the limitations of traditional metrics that evaluate acceleration and speed independently. By incorporating acceleration-speed profiles (ASPs), this method provides a more integrated interpretation of mechanical demands, accounting for the interaction between speed and acceleration. The results describe differences between forwards and backs, suggesting that metrics based on external power may offer complementary insight into positional demands compared to traditional locomotor indicators. Furthermore, analysing high external work provides practical insights into how work is distributed and the relative contributions of force, power, and speed. From an applied perspective, these findings may assist practitioners in better contextualising high-intensity locomotor efforts and interpreting training loads. However, the external power approach should be considered complementary to existing GPS-derived metrics rather than a replacement. In addition, the present framework does not account for collisions and set-piece actions, which represent a substantial component of total match load in rugby. Future research should explore the integration of locomotor and contact-based demands and further investigate the relationship between external power metrics and physiological, performance, and injury-related outcomes.

## Data Availability

The datasets presented in this article are not readily available because the dataset analysed in this study is not publicly available due to confidentiality agreements with the professional rugby team and restrictions related to the protection of sensitive performance data. These data contain information that could compromise the competitive interests and privacy of the athletes if disclosed. Data may be made available upon reasonable request to the corresponding author, subject to approval from the club and compliance with applicable data protection and ethical regulations. Requests to access the datasets should be directed to cristian.osgnach@gmail.com.
